# The Prognostic Value of Serum Creatinine Dynamics in Neonates—A Retrospective Cohort Study

**DOI:** 10.3390/jcm13237485

**Published:** 2024-12-09

**Authors:** Flavia Chisavu, Lazar Chisavu, Adalbert Schiller, Mihai Gafencu, Marioara Boia, Ramona Stroescu

**Affiliations:** 1 Centre for Molecular Research in Nephrology and Vascular Disease, Faculty of Medicine, University of Medicine and Pharmacy “Victor Babes”, Eftimie Murgu Square No. 2, 300041 Timisoara, Romania; farkas.flavia@umft.ro (F.C.); schiller.adalbert@umft.ro (A.S.); 2 “Louis Turcanu” Emergency County Hospital for Children, 300011 Timisoara, Romania; mgafencu@umft.ro (M.G.); boia.marioara@umft.ro (M.B.); stroescu.ramona@umft.ro (R.S.); 3 Discipline of Nephrology, University of Medicine and Pharmacy “Victor Babes”, Eftimie Murgu Square No. 2, 300041 Timisoara, Romania; 4 Pediatric Department, University of Medicine and Pharmacy “Victor Babes”, Eftimie Murgu Square No. 2, 300041 Timisoara, Romania; 5 Department of Obstetrics and Gynecology, University of Medicine and Pharmacy “Victor Babes”, Eftimie Murgu Square No. 2, 300041 Timisoara, Romania

**Keywords:** neonatal acute kidney injury, serum creatinine trend, mortality, chronic kidney disease, kidney impairment

## Abstract

**Background:** Acute kidney injury (AKI) is common in neonates with increased mortality and longer hospitalization. Few studies have evaluated AKI outcomes in relation to serum creatinine dynamics in neonates from the first day of life. **Methods:** We performed an observational, retrospective, single-center study on newborns admitted to the “Louis Turcanu” Emergency County Hospital for Children between 2014 and 2022. The cohort comprised 1106 neonates with their serum creatinine values recorded on the first day of life and at least another measurement taken at between days 2 and 7. We evaluated the outcomes of serum creatinine trends in relation to mortality, hospitalization and progression to chronic kidney disease. **Results:** Overall, 23.4% (259) of babies had an ascending trend of serum creatinine and on day 1 had higher urea levels, lower hemoglobin and thrombocytes, lower serum proteins and higher degrees of inflammation compared to the ones with descending trends. An ascending serum creatinine level trend was associated with increased neonatal AKI (nAKI) risk in the first seven days of 12.93 times and an increased overall nAKI risk of 4.07 times. Ascending creatinine trends independently increased mortality in the entire cohort by 1.92 times and by 4.65 times in the subgroup of patients without AKI. In the crude analysis, an ascending creatinine trend increased the risk of chronic kidney disease by 8.74 times and, in an adjusted model, only nAKI was an independent risk factor (8.57 times). **Conclusions:** Neonates are a high-risk population with prolonged hospitalization regardless of serum creatinine trend. Our study emphasizes the importance of monitoring serum creatinine trends in at-risk newborns, especially those with ascending serum creatinine trends in the first week of life. Only the ascending serum creatinine trend was independently associated with an increased risk of nAKI development and mortality. nAKI is a risk factor for progression to chronic kidney disease.

## 1. Introduction

Advances in nAKI assessment have been made in recent years with the use of a standardized definition [1]. However, there are several limitations in using SCr changes to assess unstable renal function, especially in preterm babies, as well as with using the urine output criteria for nAKI diagnosis in the first postnatal week [2]. The rate of decline in SCr after birth is correlated with GA and the transfer of maternal SCr [3], and reduced decline rates of SCr in the first week of life are associated with worse outcomes (longer hospital stays, prolonged mechanical ventilation and need of inotropes and diuretics) [2]. Despite the high-quality evidence of AKI impact in newborns’ outcomes [1,2,4,5], there is no consensus regarding the real kidney function estimated by SCr levels. However, SCr remains the easiest and cheapest method to assess kidney function. Several studies have shown that infants who failed to reach, by day 7 of life, a predefined SCr level had associated higher mortality rates, longer hospital stays and were at higher risk of developing nAKI after the first 7 days of life [2,6,7]. Less is known about SCr dynamics’ impact on the risk of nAKI.

Single-center studies in neonates suggest that AKI is common, especially in neonatal intensive care units (NICUs), with incidence rates ranging from 3 to 71% [8,9,10,11,12]. In 2017, the Assessment of Worldwide Acute Kidney Injury Epidemiology in Neonates (AWAKEN) study documented that approximately 30% of neonates admitted to NICUs developed AKI [13]. This first multi-center, multi-national project used a modified, standardized AKI definition widely accepted for use in neonatal settings, showing that neonatal AKI (nAKI) is not simply an incidental finding but a key event affecting mortality and hospital stay duration [13]. The nAKI definition was adapted from the Kidney Disease: Improving Global Outcomes (KDIGO) definition [14]. The hardship in defining and staging nAKI is more difficult in the absence of steady-state serum creatinine (SCr), which is mostly due to the rapid drop in postnatal SCr in term neonates or the lack of SCr drop in preterm groups, thus requiring the evaluation of SCr for thresholds and time frames across preterm groups [3]. Regardless of gestational age, nAKI is multifactorial and dependent on antenatal, intrapartum and early postnatal factors. Thus, careful consideration is required of the immature kidney with an already inherently low glomerular filtration rate (GFR) [4,6,15,16].

A standardized definition of AKI in neonates [1], further validated by AWAKEN [13] represented a step forward in identifying the short- and long-term effects of AKI on ‘growing’ kidneys. Yet, the nAKI KDIGO definition requires a baseline SCr measurement in these patients; thus, the first two days of life are more likely to be excluded due to early postnatal dynamics. For example, in the Preterm Erythropoietin Neuroprotection Trial (PENUT), in the absence of a steady-state SCr level, the baseline SCr was defined using the lowest previous value measured, not including any values measured on the birth day because of maternal SCr or the day after birth, as extremely low gestational age neonates (ELGAN) require 36–48 h to obtain a plateau in SCr [16].

Neonatal SCr derives from the mother, as was previously described [17,18,19]. As most neonatal studies focus on AKI outcomes (e.g., mortality, hospital length of stay, risk of recurrent AKI, etc.) [1,13,17,18,19,20], there seems to be a lack of studies regarding serum SCr dynamics from the first day of life in AKI development. To begin to fill this gap, we retrospectively assessed SCr trends in the first seven days of life in a mixed neonatal population. The aims of this study were to (1) identify the risk of AKI based on SCr trends from the first day of life, (2) evaluate SCr trends in relation to mortality rates and length of hospitalization, and (3) determine the risk of progression to chronic kidney disease.

## 2. Methods

### 2.1. Study Population

We conducted a single-center retrospective review of the medical records of 1343 neonates who were admitted in the neonatal department of the “Louis Turcanu” Emergency County Hospital for Children between 2014 and 2022. All the admitted newborns were transferred from external clinical maternity wards and underwent biological assessments based on their clinical state. Entry criteria included newborns with a recorded SCr level on the first day of life. SCr was measured using the kinetic Jaffe method. The study groups were divided based on SCr trend: ascending trend (AT) when SCr increased compared to the first day of life (the next consecutive serum creatinine measured between day 2 and 7 was higher compared with the one from the first day of life), and descending trend (DT) when there was a decline in SCr when compared to the SCr value from the first day of life (the next serum creatinine measured between day 2 and 7 was lower compared with the one from the first day of life). An unknown trend (UT) was considered in all patients that had only the SCr measurement from the first day of life (without at least another serum creatinine value recorded between days 2 and 7 of life). Two individual operators analyzed the data, and the trend was assessed as ascending when the following serum creatinine value was higher compared to the serum creatinine from the first day of life and descending when the serum creatinine was lower. SCr measurements were retrieved from the first 7 days of life and on days 14 and 28, respectively. We evaluated AKI occurrence in the first week and between days 8 and 28 of life in patients who had at least two SCr measurements that fulfill the nAKI definition. After excluding the patients with UT and the ones who died in the first 48 h after birth, the final cohort consisted of 1106 patients. All demographic and clinical data were assessed on the first day of life. We divided the patients by gestational age: term neonates (≥37 weeks), moderate to late preterm (32–36 weeks), very preterm (28–31 weeks), and extremely preterm (under 28 weeks). In our cohort, 444 patients had a SCr follow-up over 3 months.

The study was performed in accordance with the Ethics Code of the World Medical Association and the “Louis Turcanu” Emergency Hospital for Children’s Ethics Committee (reference number 6055/24 March 2022). The study followed the Declaration of Helsinki recommendations. The legal guardians of the patients signed an informed consent at the admittance into the hospital. The anonymity of the patients was maintained during the study protocol.

### 2.2. Selection Criterion

AKI was defined and staged using the modified neonatal KDIGO SCr criteria (Supplemental Table S1) [1]. AKI was diagnosed using the reference SCr as the lowest prior SCr value. In patients with AT, the baseline SCr was either the SCr from day one of life or the lowest SCr value reached before the AKI episode if the initial rising did not fulfill AKI criteria based on SCr. In the DT group, the baseline SCr was the lowest recorded value before the AKI episode. The KDIGO urine output criterion was not included. Chronic kidney disease (CKD) was defined as abnormalities of the kidney structure or function over 3 months with health implications [21]. The criteria used to define CKD were defined by the persistence over 3 months of the following: estimated glomerular filtration rate (eGFR) below 90 mL/min/1.73 m^2^ and/or persistence of kidney damage markers (proteinuria, albuminuria, and hematuria) and/or persistence of structural abnormalities identified by imaging (ultrasound, computer tomography, magnetic resonance imaging, and scintigraphy).

### 2.3. Outcomes

The primary outcome was to determine the risk of AKI development based on SCr trend in the first week of life and between days 8 and 28. The secondary outcome was to assess the impact of the SCr trend on mortality and hospital stay. The third outcome was to determine the influence of the SCr trend on CKD development.

### 2.4. Statistical Analysis

Data are presented as average ± standard deviation (SD) for normally distributed continuous variables, median and interquartile range for non-normally distributed variables, and percentages for discrete ones. All continuous variables were tested for normality using the Shapiro–Wilk test (a *p* value < 0.05 rejected normality). For normally distributed variables, we used an independent *t*-test or ANOVA. For non-normally distributed continuous variables, groups were compared using the Wilcoxon signed-rank test or Kruskal–Wallis test. Discrete variables were analyzed with the Chi-square test. The variables that influenced creatinine trend were identified using logistic regression models. Mortality risk factors were identified using logistic regression models. Kaplan–Meier survival analysis was performed for the risk of death and CKD development. A multivariate Cox proportional hazards models (HR) were used in addition to the Kaplan–Meier analysis to adjust for the confounding factors that influenced mortality or CKD, respectively, in the logistic regression models. The odds ratio (OR) and 95% confidence interval (95% CI) were calculated. In addition, we present Harrell’s C-index with the corresponding 95% CI. In this study, a *p*-value of 0.05 was considered the threshold for statistical significance. In the multivariable logistic regression models, we used the backward method where a variable was included if *p* < 0.05 and excluded if *p* > 0.1. Data were analyzed using MedCalc^®^ Statistical Software version 22.021 (MedCalc Software Ltd., Ostend, Belgium; https://www.medcalc.org; accessed on 10 February 2024).

There were some missing data in our cohort as follows: 0.54% for urea, 0.18% for weight, 0.18% for hemoglobin, 4.15% for serum proteins, 8.22% for procalcitonin, 0.45% for GOT, 0.45% for GPT, 13.92% for potassium, 14.01% for sodium and 13.74% for LDH. For the statistical analysis, we used no tools to complete the missing data.

## 3. Results

**Figure 1:**
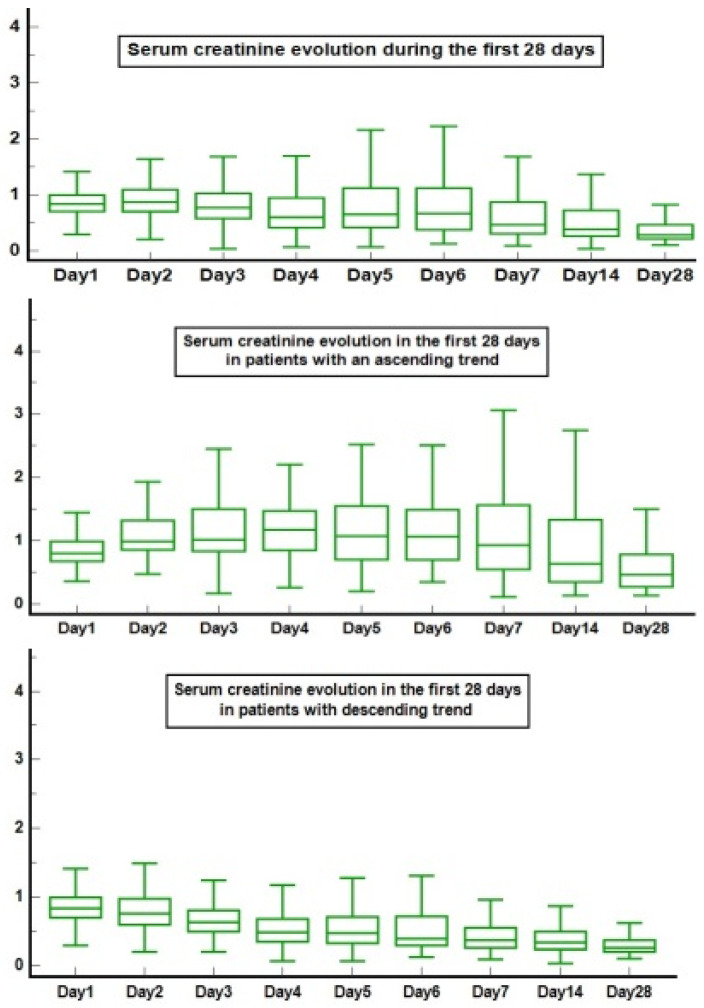
Serum creatinine dynamics during the first 28 days. Legend: on *x*-axis are the corresponding day for serum creatinine measurement, *y*-axis represents the serum creatinine values in mg/dL.

The initial cohort consisted of 1343 newborns. We excluded patients who died in the first 48 h, and the remaining cohort of 1301 was grouped based on the SCr trend: 259 babies with AT (19.9%), 847 with DT (65.1%), and 195 neonates with UT (15%). Newborns with an UT had an overall higher weight, better biological parameters, shorter admissions (including NICU), and a high mortality incidence (10.3%) (Supplemental Table S2). Further, we focused our research on patients with a known SCr trend. Thus, the final cohort comprised 1106 newborns (23.4% with an AT and 76.6% with a DT). The DT group had a higher GA, while the AT had higher incidences in lower GA categories. Out of the underlying diseases, only cardiac and renal malformations and Human Immunodeficiency Virus (HIV) exposure presented significant statistical differences between the two groups (Table 1). Babies from the AT also had lower hemoglobin, thrombocytes, serum proteins, and higher urea, degrees of inflammation, lactate dehydrogenase and transaminases (Table 2). The SCr on day one of life did not differ; however, all the other SCr measurements were higher in the AT group (Figure 1, Supplemental Table S3).

Among the overall cohort, the incidence of AKI was 29.8%. The first week of life accounted for 61.5% of the AKI cases with almost 75% being in the AT group. Two thirds of the recorded AKI episodes after the first 7 days were from the DT group. More than half of infants with AKI stages 1 and 3 were from the AT group. Regardless of SCr trend, severe AKI (stages 2 and 3) was present in almost half the cases with nAKI (Table 3). Out of the 1106 patients, a follow-up of more than 3 months was recorded in 444 cases with a median follow-up of 14.5 months. Progression to CKD was 3.2%, being three times higher in the AT group. The overall length of hospital stay (LOS) was similar between the two groups even though the AT group had a higher NICU admission with longer NICU stay. Mortality was four times higher in the AT group (29.3% compared to 7.1% in DT group). Overall, the mortality rate was significantly higher in newborns with AKI: 30.6% compared to 4.5% in non-AKI patients. Mortality rates increased with AKI severity from 20.2% in stage 1, 26.7% in stage 2, and 45.2% in stage 3 (*p* < 0.0001).

The next step in our analysis was to identify the factors that influenced the AT of SCr (Table 4). We performed a logistic regression adjusted for the first day SCr and GA. Patients with associated renal malformations were more likely to present with a rising SCr trend (OR 20.54, 95% CI 3.22–130.91, *p* = 0.0014), which was followed by NICU admission (OR 2.52, 95% CI 1.06–6.01, *p* = 0.0361) and cardiac malformations (OR = 2.22, 95% CI 1.18–4.18, *p* = 0.012). Higher urea levels and lower serum proteins were associated with an increased risk of AT.

We performed several logistic regression models to identify the risk factors associated with AKI development (Table 5). After adjusting the models for environment, sex, GA category, and day one SCr, the overall risk of AKI increased 4.07 times in the presence of ascending SCr trend (95% CI 2.77–5.99), 3.83 times for admission to NICU (95% CI 1.56–9.34), and 2.91 times for associated cardiac malformations (95% CI 1.53–5.54). The factors influencing AKI occurrence in the first 7 days and after the first week of life were different. The ascending SCr trend increased the risk of AKI by 12.93 times (95% CI 8.44–19.8) in the first 7 days. NICU admission maintained a very high risk of AKI after the first 7 days (OR = 9.57, 95% CI 2.31–39.57). Unexpectedly, renal malformations influenced AKI development only after the first week of life (OR = 6.31, 95% CI 1.58–25.16). Regarding the SCr trend, we found that DT had a higher absolute number of AKI events after the first 7 days of life, but lower incidence when compared to the ascending trend (101 vs. 46 events, 11.9% vs. 17.8%). In the analysis, AT seems to become protective in AKI development after the first 7 days of life; otherwise, this could be just an incidental finding, as in the adjusted model, the confidence interval was large and close to 1 (0.393–0.993).

In our cohort, the general mortality was 12.3%. The crude OR on the risk of death was 5.44 (95% CI 3.74–7.92, *p* < 0.0001) for AT and 9.33 (95% CI 6.18–14.09, *p* < 0.0001) for AKI. The sub-analysis of patients who did not develop AKI showed that AT increased the risk of death by 6.14 times (95% CI 2.95–12.76, *p* < 0.0001). In the logistic regression model on the risk of death, we identified several independent factors (Table 6). The ascending SCr trend and AKI per se increased mortality by 1.92 times and 2.61 times, respectively. The underlying congenital disorders increased mortality by 5.1 times in the presence of cardiac malformations and by 3.36 times in patients with chromosomes alteration. Out of the biological parameters, only lower thrombocytes, higher urea and procalcitonin levels had an impact on mortality. Based on GA, extremely premature and very premature babies had a higher risk of death in our analysis by 2.73 and 2.29 times, respectively. In patients who did not develop AKI, AT independently increased mortality by 4.65 times. Cardiac malformations and chromosome alterations increased mortality by 5.24 and 8.23 times, respectively. GA categories remained an important risk factor for death, which was higher in lower GA: from 14.79 times in extremely premature babies to 7.67 times in very premature babies and 2.47 times in moderate to late preterm babies.

**Figure 2:**
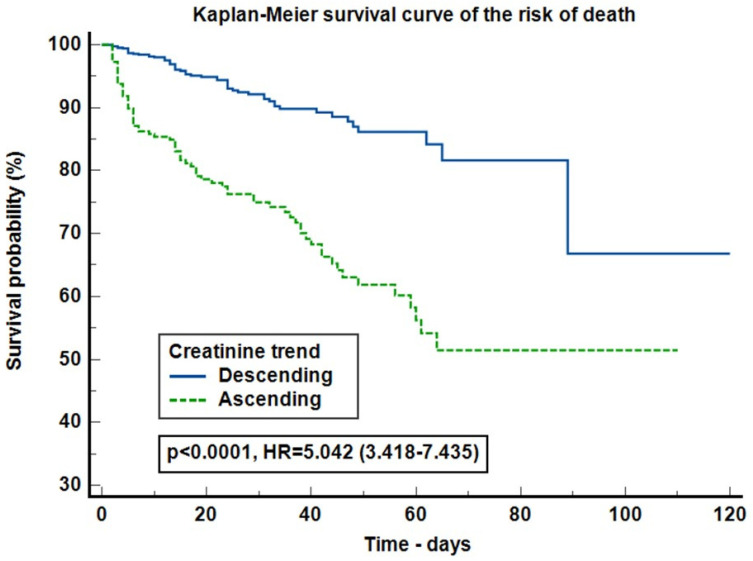
Kaplan–Meier survival curve on the risk of death. Legend: HR = hazards ratio.

**Figure 3:**
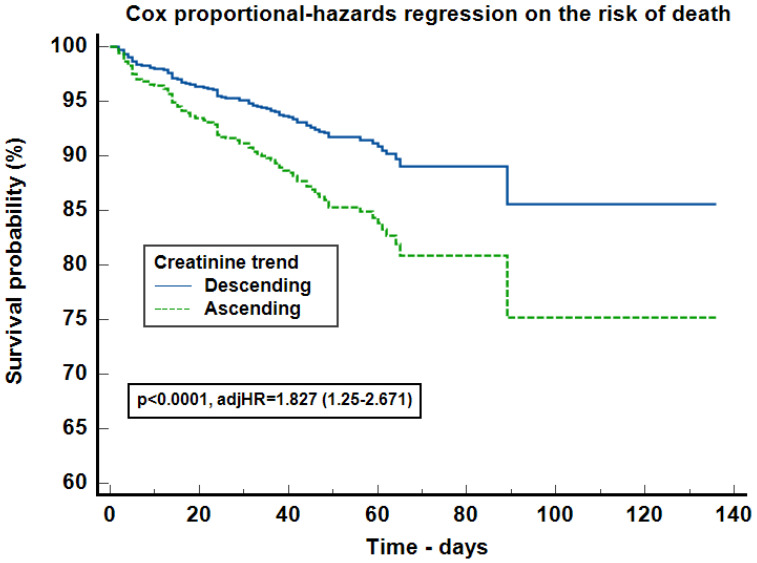
Cox proportional-hazards regression on the risk of death. Legend: adjHR = adjusted hazards ratio for acute kidney injury, procalcitonin, cardiac and digestive malformations, thrombocytes, and chromosomes alterations.

In the Kaplan–Meier survival curve on the risk of death, the number of events were 60 (7.08%) in DT group and 76 (29.34%) in AT. Ascending creatinine trend increased the risk of death by 5.042 times (95% CI: 3.418–7.435, *p* < 0.0001) compared with the decreasing creatinine trend group (Figure 2). The Cox proportional-hazards regression model (Harrell’s C-index = 0.807, 95% CI 0.767–0.847, *p* < 0.0001) on the risk of death, stratified by creatinine trend, was adjusted for: AKI, procalcitonin, cardiac and digestive malformations, thrombocytes and chromosomes alterations. The increasing SCr trend increased the risk of death by 1.827 times (95% CI: 1.25–2.671, *p* = 0.0019) (Figure 3).

**Figure 4:**
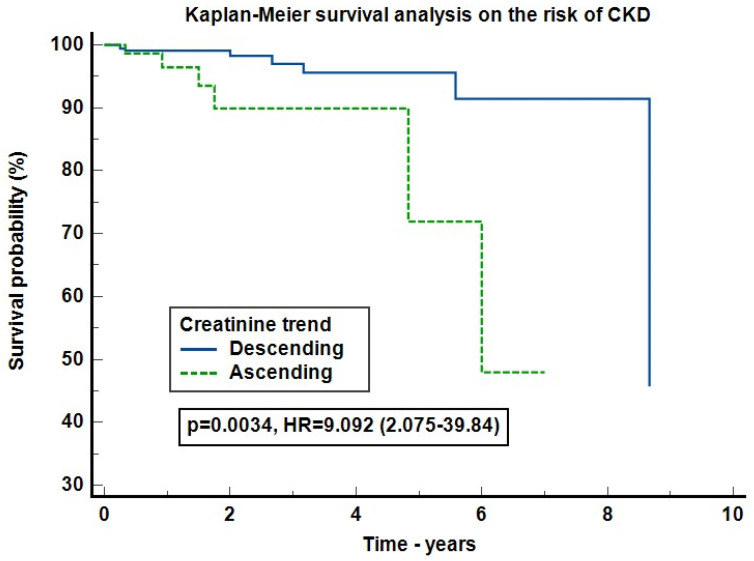
Kaplan–Meier survival analysis on the risk of CKD. Legend: The analysis was performed in the subgroup of 444 patients with a follow-up longer than 3 months, CKD = chronic kidney disease, HR = hazards ratio.

**Figure 5:**
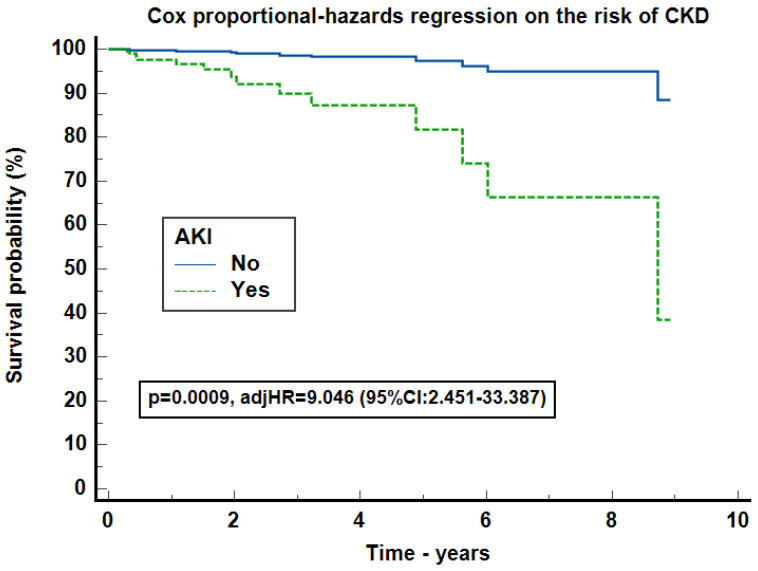
Cox proportional-hazards regression on the risk of CKD. Legend: The analysis was performed in the subgroup of 444 patients with a follow-up longer than 3 months, AKI = acute kidney injury, CKD = chronic kidney disease, adjHR = hazards ratio for serum creatinine trend, cardiac malformations, and renal malformations.

We further evaluated the risk of new-onset CKD. For this analysis, we included only patients with follow-up of more than 3 months. The cohort consisted of 444 patients with a median follow-up of 14.5 months (IQR 6.23–29.3). The Kaplan–Meyer survival curve on the risk of CKD proved to be statistical significant (*p* = 0.0034) with eight censored cases (2.37%) in the DT group and six (7.59%) in the AT one. Patients with an increasing SCr trend presented a 8.74 times higher risk of developing CKD compared to the decreasing trend ones (95% CI: 2.01–38.02, *p* = 0.0038) (Figure 4). In the Cox-proportional hazards regression on the risk of CKD, adjusted for AKI development, cardiac malformations, renal malformations and creatinine trend, the SCr trend did not influenced CKD development, but AKI increased CKD development by 9.046 times (95% CI: 2.451–33.387), *p* = 0.0009. The model had a significance level of *p* = 0.0002 with Harrell’s C-index = 0.765 (95% CI: 0.612–0.918) (Figure 5).

## 4. Discussions

Given the unstable kidney function in newborns, we evaluated the serum creatinine trend from the first day of life to see if an ascending or descending trend impacts mortality and hospitalization. The main objective was to establish the link between rising serum creatinine levels and the risk of AKI in the first week of life. Once the serum creatinine trend is rising, the risk of AKI increases exponentially, leading to an increased risk of progression to CKD. Mortality was higher in the AT regardless of the serum creatinine rise.

The natural dynamics of the SCr levels after birth should be represented by a linear decrease in the first seven days, even longer in lower GA [12,19,22], as seen in the majority of the admitted patients from our cohort (76.6%)—Table 1. However, we did not find differences regarding the first recorded serum creatinine in different gestational ages but rather a major difference in reaching the baseline serum creatinine level over time that was dependent of the AT of serum creatinine—Supplemental Table S3 and Figure 1. With this study, we underline the importance of repeated functional renal tests in at-risk newborns.

Our overall AKI incidence was similar to previously published data (29.8%) (Table 3) [1]. Patients from the AT group had similar SCr levels in the first day of life but higher values in all following days, including day 28. The AT group showed extremely high overall AKI occurrence (70%), mostly in the first 7 days of life (58.7%) (Table 3). We identified several risk factors that increased the risk of an ascending serum creatinine trend: renal malformations, cardiac malformations, and NICU admission (Table 4). Infants from the AT group presented with worse biological parameters in the first day of life. Yet, only higher levels of urea and lower serum proteins had an independent impact on SCr trend. We justify that these infants have poorer outcomes based on clinical and biological assessments, mirroring the severity of the underlying disease. Thus, kidney impairment is a consequence of a multifactorial spectrum of risk factors that further becomes an independent risk factor for worse outcomes, even in small rises of SCr.

Associated cardiac malformations remained a risk factor for overall AKI occurrence while renal malformations only had an impact on AKI development after 7 days (Table 5). Surprisingly, the GA category did not statistically influence AKI occurrence. This is most likely a consequence of overlapping GA categories over the other cofounders in the regression model. Nevertheless, AKI severity and incidence increase in lower GA. However, this is to be expected, as prematurity translates into an underdeveloped kidney, which is more susceptible to kidney injury.

More than 80% of neonates required NICU admission, which increased the risk of overall AKI and AKI after the first 7 days (Table 5). Surely, in the NICU settings, these patients have a higher risk for AKI occurrence besides the intra and peripartum events and more often are exposed to high-risk medical interventions (e.g., mechanical ventilation, use of nephrotoxic medications, inotropes or nosocomial infections) [5]. In addition, patients who required NICU admission presented a more severe underlying disease. NICU admission meant higher LOS regardless of the SCr trend.

Neonatal mortality rates has decreased over the last several years [23,24]. Evidence-based medicine currently recognizes AKI as an independent mortality risk factor [1,3,4,5,25]. In addition to the high mortality rates associated with AKI, we also found the ascending SCr trend to be an independent mortality risk factor (Table 6, Figure 2 and Figure 3). With these results, we underline that besides the traditional known factors that increase mortality (cardiac malformations, mechanical ventilation, nephrotoxic exposure, AKI, etc.), kidney impairment, not necessary injury, translated in small rises of SCr levels, is associated with worse outcomes. Although kidney impairment and AKI proved to influence survival, they represent different sides of the same coin.

Unlike other pediatric age groups, neonatal renal physiology is poorly understood, especially in those who did not reach the theoretical landmark of nephron development (week 34–36 of gestation) [26]. Our current evaluation method using the SCr trend in an immature kidney could be a red flag for clinicians at the bedside to assess kidney function repeatedly and to identify aggressors for proper management. We propose the SCr trend be used in clinical practice, as it is easier to implement on a large scale in neonatal settings (including NICU) as a complementary tool to the current nAKI definition.

The highlight of our study was the analysis of the subgroup of patients without AKI. Even in the absence of AKI, kidney impairment can evolve toward AKI or it can associate a decline in SCr that, afterward, can or cannot progress to AKI, or will continue a linear decline until reaching the nadir.

So far, newborns born prematurely have been at higher risk for CKD development [15]. However, there is a lack of evidence that nAKI patients have an increased risk of CKD, as most of them are lost to follow-up. In the absence of an underlying renal pathology (congenital kidney and urinary tract malformations, genetic disorders, etc.), evolution to CKD is mostly linked to the reduced nephron number. In our study, only 4 out of 10 neonates had a SCr follow-up of more than 3 months. CKD prevalence was low (3.2%), and only patients with AKI had a higher risk of CKD development (Figure 4 and Figure 5). CKD development is a multifactorial outcome of several risk factors besides AKI. Some of these factors are common to both CKD and AKI development (prematurity, renal malformations, cardiac malformations, etc.). Therefore, attention should focus on patients at risk with closer follow-up and early intervention.

Keeping in mind that SCr is a delayed marker of kidney function (48–72 h) that reflects the function, not the injury, as it is dependent of the muscle mass, and it requires a longer time to achieve the nadir in neonates, careful interpretation is required [4,5,16]. Our results prove that once the trend is rising, infants require more than 28 days to reach the nadir.

The single center, the retrospective nature, lack of urine output criteria for nAKI, the reduced follow-up of SCr later in life, and the lack of nephrotoxic exposure represent the major limitations of our study. Another major limitation was the non-inclusion of the diagnostic for the transfer from the outer maternity wards. The strengths are the high number of patients, the serum creatinine dynamics recorded from the first 7 days of life, on days 14 and 28, respectively, and the risk of CKD development in neonates. In addition, to our knowledge, this is the first study to evaluate the SCr trend from the first day of life and the impact of rising SCr levels with or without AKI. Also, this is the first time that CKD development is explored in neonates following the SCr trend. To validate our results, there is an imperative need for multi-center studies on the impact of serum creatinine dynamics in neonates.

## 5. Conclusions

Regardless of gestational age, the serum creatinine levels in the first week of life should follow a linear decrease. In our cohort, one out of four admitted neonates had an ascending SCr trend that was further associated with a higher incidence of AKI occurrence. The mortality risk increased in the presence of small rises in SCr levels, even outside the nAKI definition. NAKI proved to increase the risk of CKD development.

## Figures and Tables

**Table 1:** Demographics and clinical characteristics.

Variable		Ascending Trend N = 259 (23.4%)	Descending Trend N = 847 (76.6%)	Total N = 1106	*p*-Value
Gender—male		161 (62.2%)	473 (55.8%)	634 (57.3%)	0.054 ^1^
Environment—urban		168 (64.9%)	512 (60.4%)	680 (61.5%)	0.2014 ^1^
Weight (grams) M + IQR		2450 (1540–3030)	2540 (1900–3080)	2520 (1800–3060)	0.0126 ^2^
GA weeks		36 (32–38)	37 (34–38)	36 (34–38)	0.0001 ^1^
GA groups	<28 weeks	21 (8.1%)	27 (3.2%)	48 (4.3%)	<0.0001 ^1^
	28–31 weeks	43 (16.6%)	76 (9%)	119 (10.8%)	
	32–36 week	83 (32%)	307 (36.2%)	390 (35.3%)	
	>36 weeks	112 (43.2%)	437 (51.6%)	549 (49.6%)	
Gemelarity		21 (8.1%)	65 (7.7%)	86 (7.8%)	0.8195 ^1^
Cardiac malformations		25 (9.7%)	40 (4.7%)	65 (5.9%)	0.0032 ^1^
Digestive malformation		35 (13.5%)	124 (14.6%)	159 (14.4%)	0.6513 ^1^
Chromosome alterations		10 (3.9%)	21 (2.5%)	31(2.8%)	0.2386 ^1^
Neurological malformations		13 (5%)	37 (4.4%)	50 (4.5%)	0.6592 ^1^
Renal malformations		7 (2.7%)	3 (0.4%)	10 (0.9%)	0.0005 ^1^
HIV exposure		3 (1.2%)	90 (10.6%)	93 (8.4%)	<0.0001 ^1^

Legend: ^1^ Chi-square test, ^2^ Mann–Whitney test, GA = gestational age, N = number, M + IQR = median and interquartile range, HIV = human immunodeficiency virus.

**Table 2:** Biological parameters from the first day of life.

Variable	Ascending Trend N = 259 (23.4%)	Descending Trend N = 847 (76.6%)	Total N = 1106	*p* Value
Day 1 SCr (µmol/L) M + IQR	0.8 (0.67–0.99)	0.83 (0.7–1)	0.83 (0.7–1)	0.2399 *
Maximum SCr (mg/dL) M + IQR	1.41 (0.97–2.16)	0.85 (0.72–1.05)	0.91 (0.75–1.23)	<0.0001 *
Minimum SCr (mg/dL) M + IQR	0.38 (0.23–0.63)	0.26 (0.2–0.37)	0.28 (0.2–0.38)	<0.0001 *
Urea (mmol/L) M + IQR	19.5 (9.91–28.65)	6.19 (3.93–11.35)	7.67 (4.38–15.51)	<0.0001 *
Hemoglobin (g/dL) M + IQR	9.4 (7.7–11.95)	11.8 (9–14.8)	11.1 (8.6–14.4)	<0.0001 *
Thrombocytes (N/mmc) M + IQR	163,000 (78,000–242,750)	213,000 (141,750–277,250)	205,000 (129,500–274,000)	<0.0001 *
Serum proteins (g/L) M + IQR	43.4 (38.3–47.4)	47.6 (43.2–51.4)	46.6 (41.9–50.8)	<0.0001 *
C reactive protein (mg/dL) M + IQR	11.83 (2.38–50.18)	3.52 (0.93–19.78)	4.46 (1.11–24.6)	<0.0001 *
Procalcitonin (ng/mL) M + IQR	10.41 (3.06–33.58)	4.01 (1.15–16.24)	5.19 (1.41–20.39)	<0.0001 *
GOT (U/L) M + IQR	67 (40–120.5)	54 (38–79)	57 (38–86)	<0.0001 *
GPT (U/L) M + IQR	21 (9–68)	14 (9–29)	14 (9–35)	<0.0001 *
K (mmol/L) M + IQR	5.2 (4.5–5.82)	5.1 (4.5–5.7)	5.1 (4.5–5.7)	0.2823 *
Na (mmol/L) M + IQR	134 (131–138)	135 (132–137)	135 (132–137)	0.0571 *
LDH (U/L) M + IQR	839.5 (618–1346.5)	677 (511–951)	721 (527–1137)	<0.0001 *

Legend: Parameters presented as mean + IQR; * Mann–Whitney test, N = number, µmol = micromoles, L = liter, M + IQR = median and interquartile range, mmol = millimol, g = gram, dL = deciliter, mmc = cube millimeter, mg = milligram, mL = milliliter, U = units, GOT = glutamic–oxaloacetic transaminase, GPT = glutamic–pyruvic transaminase, LDH = lactate dehydrogenase, K = potassium, Na = sodium.

**Table 3:** Outcomes of serum creatinine trend.

Variable		Ascending Trend N = 259 (23.4%)	Descending Trend N = 847 (76.6%)	Total N = 1106	*p* Value
AKI in the first 7 days		152 (58.7%)	51 (6%)	203 (18.4%)	<0.0001 *
AKI after the first 7 days		46 (17.8%)	101 (11.9%)	147 (13.3%)	0.0155 *
Overall AKI		181 (69.9%)	149 (17.6%)	330 (29.8%)	<0.0001 *
AKI stage	Stage 1	76 (29.3%)	53 (6.3%)	129 (11.7%)	<0.0001 *
	Stage 2	41 (15.8%)	45 (5.3%)	86 (7.8%)	
	Stage 3	64 (24.7%)	51 (6%)	115 (10.4%)	
CKD		6/88 (6.8%)	8/356 (2.2%)	14/444 (3.2%)	0.0282 *
Hospital stay days M + IQR		24 (11–43)	19 (11–35)	20 (11–37)	0.0645 ^2^
NICU admission		251 (96.9%)	682 (80.5%)	933 (84.35%)	<0.0001 *
NICU days ^1^ M + IQR		10 (5–18)	7 (4–13)	7 (4–14)	<0.0001 ^2^
Deaths		76 (29.3%)	60 (7.1%)	136 (12.3%)	<0.0001 *

Legend: Data are presented as median and IQR; * Chi-square test; ^1^ Only for NICU admissions; ^2^ Mann–Whitney test, AKI = acute kidney injury; CKD = chronic kidney disease; NICU = neonatal acute intensive care, M + IQR = median and interquartile range.

**Table 4:** Logistic regression for factors that influence the serum creatinine—ascending trend.

Variable	Odds Ratio	95% CI	*p* Value
Serum proteins (g/L)	0.93	0.902–0.958	<0.0001
Cardiac malformations	2.229	1.187–4.183	0.0126
Renal malformations	20.548	3.225–130.913	0.0014
ICU admission	2.528	1.061–6.019	0.0361
Urea (mmol/L)	1.101	1.081–1.122	<0.0001

Legend: CI = confidence interval, AUC = 0.834 (95% CI = 0.809–0.857), *p* < 0.0001, Nagelkerke R square = 0.335. AUC = are under the curve, g = grams, L = liter, mmol = millimol. Model adjusted for first day creatinine and gestational age.

**Table 5:** Logistic regressions for factors that influenced AKI occurrence.

Variable	AKI in the First 7 Days	AKI After 7 Days	Overall AKI
Creatinine trend (ascending)	12.933 (8.446–19.804)	0.625 (0.393–0.993)	4.079 (2.773–5.999)
Urea mmol/L	1.093 (1.073–1.114)	1.038 (1.019–1.056)	1.134 (1.108–1.162)
Hemoglobin g/dL	Excluded	0.921 (0.866–0.979)	0.944 (0.895–0.997)
Thrombocytes/1000/mmc	Excluded	0.998 (0.996–1.0002)	0.998 (0.996–0.999)
NICU admission (yes)	Excluded	9.573 (2.315–39.579)	3.830 (1.569–9.348)
Cardiac malformations (yes)	Excluded	1.724 (0.904–3.287)	2.917 (1.534–5.548)
Renal malformations (yes)	Excluded	6.319 (1.587–25.162)	Excluded
Day 1 SCr mg/dL	1.918 (1.042–3.529)	0.354 (0.183–0.682)	0.462 (0.275–0.776)
Gestational age category	Very preterm: 1.753 (0.964–3.186)	Excluded	Excluded
AUC	0.915 (0.897–0.930)	0.75 (0.723–0.775)	0.885 (0.864–0.903)
Nagelkerke R square	0.540	0.159	0.53

Legend: Values expressed as odds ratio and 95% confidence interval. Models adjusted for day 1 serum creatinine, gestational age and environment. SCr = serum creatinine, AKI = acute kidney injury, ICU = neonatal intensive care unit, AUC = area under the curve, mmol = millimols, L = liter, g = grams, dL = deciliter, mmc = cube millimeter.

**Table 6:** Logistic regressions for factors that influence mortality.

Variable	All Patients	Non-AKI Patients
Creatinine trend (ascending)	1.925 (1.171–3.164)	4.654 (1.988–10.895)
AKI (yes)	2.615 (1.509–4.533)	-
Procalcitonin ng/mL	1.012 (1.005–1.019)	1.013 (1.0006–1.026)
Cardiac malformations (yes)	5.108 (2.395–10.891)	5.248 (1.168–23.565)
Urea mmol/L	1.019 (0.997–1.04)	Excluded
Thrombocytes/1000/mmc	0.990 (0.987–0.993)	0.991 (0.987–0.996)
Digestive malformations (yes)	1.750 (0.899–3.405)	Excluded
Chromosomes alterations (yes)	3.368 (1.24–9.145)	8.231 (1.655–40.923)
Gestational age <28 weeks	2.732 (1.205–6.192)	14.79 (3.282–66.645)
Gestational age 28–31 weeks	2.295 (1.238–4.251)	7.670 (2.525–23.294)
Gestational age 32–36 weeks	Excluded	2.471 (0.922–6.616)
AUC	0.886 (0.865–0.905)	0.835 (0.805–0.862)
Nagelkerke R square	0.439	0.287

Legend: Values are expressed as odds ratio and 95% confidence interval. For gestational age categories, the baseline was the gestational age higher than 36 weeks. AUC = are under the curve, AKI = acute kidney injury, ng = nanograms, mL = milliliter, mmol = millimol, L = liter, mmc = millimeter cube.

## Data Availability

All the anonymized data can be available at the specific request to the corresponding author at e-mail: chisavu.lazar@umft.ro. After agreement from all the authors and after the acceptance of the request by the Ethics Committee from Emergency Hospital for Children “Louis Turcanu” from Timisoara, the anonymized data will be shared. We did not upload the data due to security considerations.

## Supplementary Materials

The following supporting information can be downloaded at https://www.mdpi.com/article/10.3390/jcm13237485/s1, Supplemental Table S1: Neonatal AKI KDIGO Classification. Supplemental Table S2: Baseline characteristics. Supplemental Table S3: Serum creatinine evolution during first 7 days, day 14 and day 28.
