# Supplementary material for: The Prognostic Value of Serum Creatinine Dynamics in Neonates—A Retrospective Cohort Study
Source: J Clin Med. 2024 Dec 9;13(23):7485. doi: 10.3390/jcm13237485 (PMC11642538; doi:10.3390/jcm13237485)
Supplement: Supplementary file 1 [file jcm-13-07485-s001.zip › jcm-3342071-supplementary.pdf]

Supplemental Table 1 - Neonatal AKI KDIGO Classification

Stage 0	No change in SCr level or rise <0.3mg/dl
Stage 1	Increase in SCr level of ≥0.3mg/dl within 48 hours or rise in SCr level ≥ 1.5 – 1.9 times the reference SCr level within 7 days
Stage 2	Rise in SCr level ≥ 2 – 2.9 times the reference SCr level within 7 days
Stage 3	SCr level ≥ 3 times the reference SCr level or SCr > 2.5mg/dl or receipt of RRT

Legend: SCr=serum creatinine; reference SCr=the lowest previous SCr value; RRT=renal replacement therapy

Supplemental Table 2 Baseline characteristics

Variable	Ascending trend N=259	Descending trend N=847	Unknown trend N= 195	Total N=1301	P value
Day 1 SCr mg/dl M+IQR	0.8 (0.67-0.99)	0.83 (0.7-1)	0.68 (0.61-0.8)	0.8 (0.67-0.97)	<0.0001[1]
Maximum SCr mg/dl M+IQR	1.41 (0.97-2.16)	0.85 (0.72-1.05)	-	0.91 (0.75-1.23)	<0.0001[2]
Minimum SCr mg/dl M+IQR	0.38 (0.23-0.63)	0.26 (0.2-0.37)	-	0.28 (0.2-0.41)	<0.0001[2]
Urea mmol/l M+IQR	19.5 (9.91-28.65)	6.19 (3.93-11.35)	3.71 (2.91-4.7)	6.4 (3.9-13.63)	<0.0001[1]
Haemoglobin g/dl M+IQR	9.4 (7.7-11.95)	11.8 (9-14.8)	15.1 (12.77-16.6)	11.8 (8.9-15.1)	<0.0001[1]
Thrombocytes N/cmm M+IQR	163000 (78000-242750)	213000 (141750-277250)	252000 (190750-309250)	212000 (136000-279250)	<0.0001[1]
Serum proteins g/dl M+IQR	43.4 (38.3-47.4)	47.6 (43.2-51.4)	47.5 (43.05-51.37)	46.7 (42.1-50.9)	<0.0001[1]
C reactive protein mg/l M+IQR	11.83 (2.38-50.18)	3.52 (0.93-19.78)	1.21 (0.49-4.47)	3.59 (0.87-20.49)	<0.0001[1]
Procalcitonin ng/ml M+IQR	10.41 (3.06-33.58)	4.01 (1.15-16.24)	2.73 (0.97-8.16)	4.76 (1.26-18.42)	<0.0001[1]
GOT U/l M+IQR	67 (40-120.5)	54 (38-79)	57 (40-75.5)	57 (38.75-84)	<0.0001[1]
GPT U/l M+IQR	21 (9-68)	14 (9-29)	10.5 (6.5-17)	14 (8-30.25)	<0.0001[1]
K mmol/l M+IQR	5.2 (4.5-5.82)	5.1 (4.5-5.7)	4.85 (4.5-5.4)	5.05 (4.5-5.7)	0.0142[1]
Na mmol/l M+IQR	134 (131-138)	135 (132-137)	135 (133-137)	135 (132-137)	0.057[1]
LDH U/l M+IQR	839.5 (618-1346.5)	677 (511-951)	712 (533.25-915.25)	718 (529.25-989)	<0.0001[1]
Gender - male	161 (62.2%)	473 (55.8%)	118 (60.5%)	752 (57.8%)	0.097[3]
Environment - urban	168 (64.9%)	512 (60.4%)	133 (68.2%)	816 (62.5%)	0.028[3]
Hospital stay-days M+IQR	24 (11-43)	19 (11-35)	14 (9-27)	19 (10.75-35)	<0.0001[1]
ICU admission	251 (96.9%)	682 (80.5%)	151 (77.4%)	1084 (83.3%)	<0.0001[3]
NICU stay-days (only for NICU admissions) M+IQR	10 (5-18)	7 (4-13)	4 (3-7)	7 (4-13)	<0.0001[1]
Deaths	76 (29.3%)	60 (7.1%)	20 (10.3%)	156 (12%)	<0.0001[3]

Weight-grams M+IQR		2450 (1540-3030)	2540 (1900-3080)	2735 (1900-3280)	2560 (1820-3100)	0.0144[1]
GA weeks M+IQR		36 (32-38)	37 (34-38)	37 (34-38)	37 (34-38)	0.0001[1]
GA groups	<28 weeks	21 (8.1%)	27 (3.2%)	4 (2.1%)	52 (4%)	<0.0001[3]
	28-31 weeks	43 (16.6%)	76 (9%)	15 (7.8%)	134 (10.3%)	
	32-36 week	83 (32%)	307 (36.2%)	72 (37.3%)	462 (35.6%)	
	>36 weeks	112 (43.2%)	437 (51.6%)	102 (52.8%)	651 (50.1%)	
Gemelarity		21 (8.1%)	65 (7.7%)	18 (9.2%)	104 (8%)	0.768[3]
Cardiac malformations		25 (9.7%)	40 (4.7%)	5 (2.6%)	70 (5.4%)	0.0015[3]
Digestive malformation		35 (13.5%)	124 (14.6%)	7 (3.6%)	166 (12.8%)	0.0002[3]
Chromosome alterations		10 (3.9%)	21 (2.5%)	2 (1%)	33 (2.5%)	0.1612[3]
Neurological malformations		13 (5%)	37 (4.4%)	3 (1.5%)	53 (4.1%)	0.1361[3]
Renal malformations		7 (2.7%)	3 (0.4%)	0 (0%)	10 (0.8%)	0.0003[3]
HIV exposure		3 (1.2%)	90 (10.6%)	13 (6.7%)	106 (8.1%)	<0.0001[3]

Legend: [1] : Kruskal-Wallis test, [2] : Mann-Whitney test, [3] : Chi-square test, N=number, SCr=serum creatinine, M+IQR=median and interquartile range, mg=milligrams, dl=decilitre, mmol=millimols, l=litre, g=grams, cmm=cube millimetre, ng=nanograms, U=units, GOT= glutamic-oxaloacetic transaminase, GPT= glutamic-pyruvic transaminase, LDH= lactate dehydrogenase, K=potassium, Na=sodium, NICU=neonatal intensive care unit, GA=gestational age.

Supplemental Table 3 Serum creatinine evolution during the first 7 days, day 14 and day 28

Variable	AT group		DT group		*p* value*
	N	Median	N	Median	
SCr day 1	259	71.0000	847	74.0000	0.2399
SCr day 2	102	87.5000	149	67.0000	<0.0001
SCr day 3	150	90.0000	233	56.0000	<0.0001
SCr day 4	99	104.0000	233	43.0000	<0.0001
SCr day 5	107	95.0000	151	42.0000	<0.0001
SCr day 6	76	94.5000	105	35.0000	<0.0001
SCr day 7	148	82.5000	290	33.0000	<0.0001
SCr day 14	178	56.5000	363	30.0000	<0.0001
SCr day 28	125	41.0000	346	23.0000	<0.0001

Legend: AT=ascending SCr trend; DT=descending SCr trend; N=number, SCr= serum creatinine, *Mann-Witney test
